# 1205. Vaccine Hesitancy in Paediatric Practice and Predictors of Physician-Reported Vaccine Compliance

**DOI:** 10.1093/ofid/ofab466.1397

**Published:** 2021-12-04

**Authors:** Kate E Allan

**Affiliations:** University of Toronto, Toronto, Ontario, Canada

## Abstract

**Background:**

This study explores the frequency with which Canadian paediatricians encounter vaccine hesitancy in their clinical practice, the most common approaches to parent resistance, impact of hesitancy practice and predictors of physician-reported vaccine compliance.

**Methods:**

This analysis used data collected from Canadian paediatricians and paediatric subspecialists via a one-time survey distributed by the Canadian Paediatric Surveillance Program in the fall of 2015. Descriptive analyses were conducted to determine the frequency of hesitancy, approaches to parent resistance and impact on clinical practice. A classification tree was generated to determine the most important predictors of physician-reported vaccine compliance.

**Results:**

A total of 669 paediatricians completed the survey. Eighty-nine percent (n=588) of respondents indicated they had encountered hesitancy in their practice, with the top concerns including: Autism, too many vaccines, risk of a weakened immune system, and vaccine additives. The most common responses to parent resistance included discussing risks of non-vaccination, restating the vaccine recommendation and referring to reliable patient resources. Forty-five percent (n=301) of physicians indicated that hesitancy impacted their practice. Overall, the best predictor of physician-reported vaccine compliance was the use of a personal endorsement or anecdote (x2=6.955,df=1, adj.p< 0.01). Among physicians who did not use a personal endorsement, the next best predictor of vaccine compliance was spending 10 minutes or more discussing vaccination (x2=7.418, df=1,adj.p< 0.05).

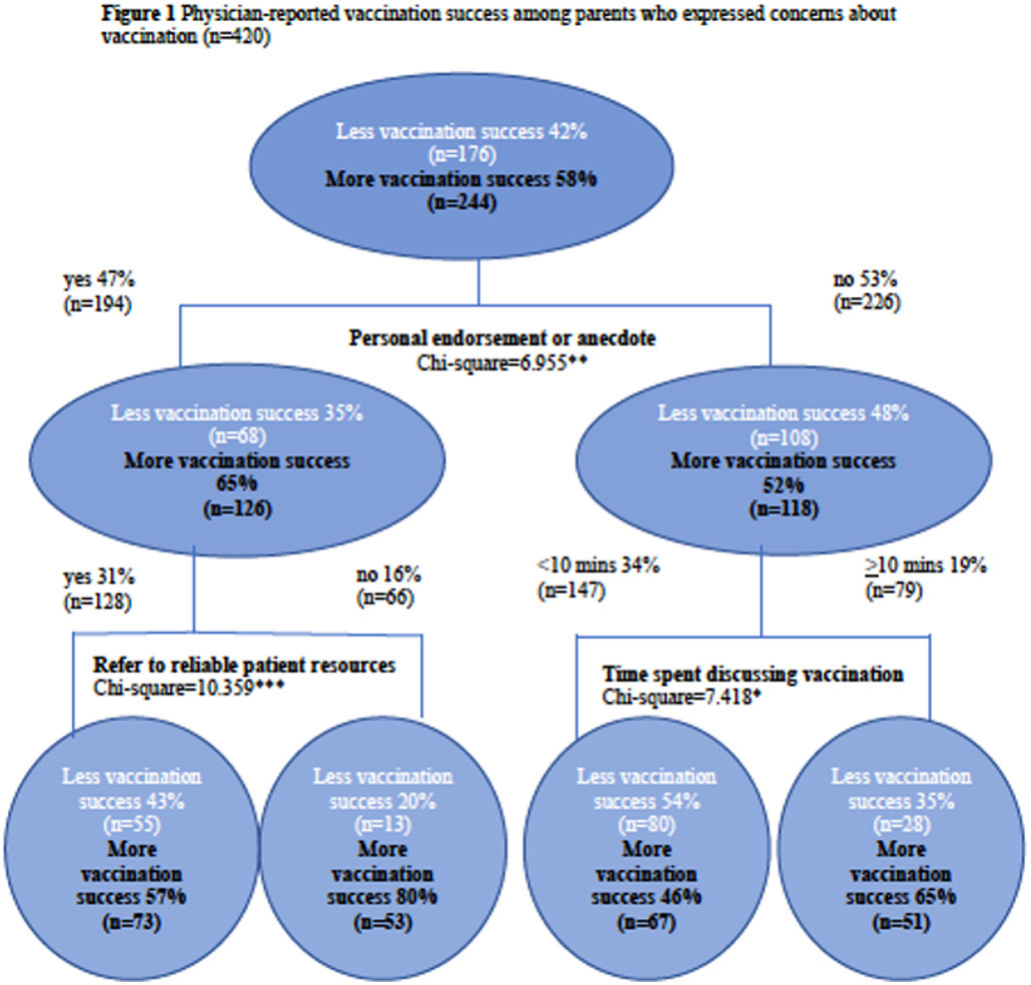

**Conclusion:**

This study contributes to a nascent body of literature related to paediatricians’ experience with vaccine hesitancy in a Canadian context, particularly as it relates to the impact of hesitancy on practice. This study demonstrates the ubiquity of hesitancy in clinical practice, the profound impact of hesitancy on paediatricians and highlights promising responses to parental hesitancy that may improve vaccine compliance. Future research should explore potential hesitancy interventions including using a personal endorsement and prolonged engagement using more rigorous methods of evaluation.

**Disclosures:**

**Kate E. Allan, PhD**, **Pfizer** (Other Financial or Material Support, Postdoctoral Fellowship at the Centre for Vaccine-Preventable Diseases (at University of Toronto) is funded by Pfizer.)

